# Secondary hemochromatosis as a result of acute transfusion-induced iron overload in a burn patient

**DOI:** 10.1186/s41038-016-0034-z

**Published:** 2016-05-02

**Authors:** Michael Amatto, Hernish Acharya

**Affiliations:** 1 University of Alberta, 8440 112 Street, Edmonton, AB T6G 2B7 Canada; 2 Glenrose Rehabilitation Hospital, #1126, 10230 111 Ave, Edmonton, AB T5G 0B7 Canada

**Keywords:** Secondary hemochromatosis, Transfusion, Iron overload, Burn patients

## Abstract

**Background:**

Red blood cell transfusions are critical in burn management. The subsequent iron overload that can occur from this treatment can lead to secondary hemochromatosis with multi-organ damage.

**Case Presentation:**

While well recognized in patients receiving chronic transfusions, we present a case outlining the acute development of hemochromatosis secondary to multiple transfusions in a burn patient.

**Conclusions:**

Simple screening laboratory measures and treatment options exist which may significantly reduce morbidity; thus, we believe awareness of secondary hemochromatosis in those treating burn patients is critical.

## Background

Acute and chronic treatment of the severely burned individual is often complex due to many physical and psychological factors [1, 2]. Resuscitation involving packed red blood cell (RBC) transfusion is often essential [3]. However, RBC transfusion carries potential risks including hemolytic reactions and infections, as well as other complications that are often overlooked such as iron overload [4, 5]. Each unit of RBCs contains 200–250 mg of iron, and with no physiologic excretion mechanism, multiple transfusions can result in iron overload [6]. Iron overload is not benign and can result in the development of life threatening chronic disease processes.

While iron overload can be caused by RBC transfusions, there is a wide differential for this abnormality that can be divided based on heritability. The hereditary category includes hemochromatosis gene (HFE) and non-HFE-related genetic defects both leading to an abnormality of increased absorption of dietary iron. Non-hereditary causes of iron overload include repeat RBC transfusions to treat chronic anemia in diseases such as thalassemia major, chronic hemolytic anemia and others, chronic liver disease, iatrogenic administration of parental iron or RBC transfusions, neonatal iron overload, aceruloplasminemia, and African iron overload [6, 7].

Hemochromatosis is a disease characterized by iron accumulation in tissues. Initial symptoms and signs include skin pigmentation, fatigue, erectile dysfunction, and arthralgia while later stages of the disease result in cardiomyopathy, diabetes mellitus, hypogonadism, hypopituitarism, and hypoparathyroidism, as well as liver fibrosis and cirrhosis which can lead to hepatocellular carcinoma [6]. As iron overload is the initial step to developing hemochromatosis, the differential diagnosis for such is the same as we have described for iron overload. Here, we present the first reported case in the literature, to the best of our knowledge, of secondary hemochromatosis from parenteral iron overload due to multiple RBC transfusions in a burn patient.

## Case presentation

A previously healthy 19-year-old male was transferred to hospital with extensive deep burns covering approximately 85 % total body surface area (TBSA). After initial assessment and debridement in the operating room, he was brought to the general systems intensive care unit (ICU) for acute renal failure likely due to myoglobulinuria. He remained in the ICU for 34 days until he was transferred to the burn unit. Several operations including multiple debridements and allograft applications were performed throughout his time in the ICU and burn unit. During his extended stay, he was treated for many complications, including multiple infections, pancreatitis, deep vein thrombosis, pleural effusion, cardiomyopathy, chronic diarrhea, depression, as well as upper airway and upper gastrointestinal tract bleeding secondary to ulcers. As well, he developed another acute kidney injury thought to be secondary to acute tubular necrosis and had multiple instances of elevated liver enzymes and abnormal liver function tests. Both of these organ insults were thought to be secondary to medications administered, as these values corrected with discontinuation of these insulting agents. In total, his acute care admission lasted approximately 15 months with 33 operations/procedures needed before he could be transferred to a rehabilitation unit.

Throughout his prolonged stay, he was worked up for anemia as he required massive amounts of RBC transfusions to maintain an average hemoglobin value over his admission of 95 g/L. This value fluctuated regularly falling into the low 70s on many occasions necessitating the use of blood products. Multiple gastrointestinal bleeds presenting with melena were thought to be large contributors to this laboratory abnormality. The question of hemolysis was raised as early on in his treatment course; he occasionally had elevated lactate dehydrogenase and bilirubin values, as well as positive direct antiglobulin tests. However, his haptoglobin never fell below the normal range and multiple peripheral blood smears had minimal morphological evidence of hemolysis. His chronic anemia was managed with multiple RBC transfusions throughout his hospital admission.

**Fig. 1:**
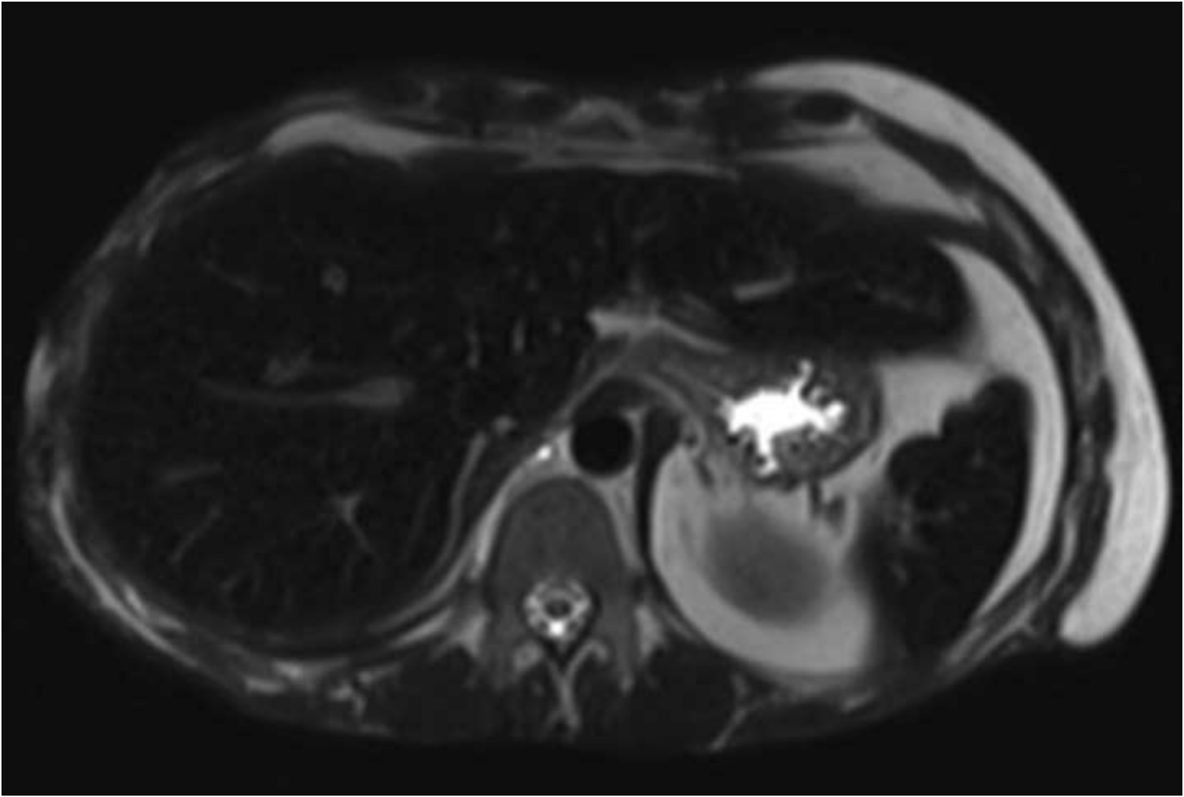
Transfusion-induced iron overload. Axial T2 HASTE TE 90 sequence showing the liver and spleen with low signal absorption caused by iron saturation. Image obtained from the Alberta Netcare Portal

While in hospital, he had multiple zinc, selenium, and copper serum values in the low and normal ranges. However, he was noted to have elevated serum iron levels and an increased iron saturation index reaching 94 % (normal <60 %). His iron indices were followed throughout his stay as all values progressed to outside the normal limits (Table 1). Subsequent magnetic resonance imaging (MRI) of the abdomen verified the diagnosis of hemochromatosis by showing iron deposition in the liver and spleen (Fig. 1). A search for etiology revealed that in just less than 15 months, he had received 292 units of packed RBCs. He did not receive any exogenous administration of iron during his treatment course. With this information, it was thought that the increased iron saturation was due to a transfusional iron overload. Given his ethnicity and age in the presence of a clear alternative diagnosis, the clinical decision to forego genetic testing was made by the Internal Medicine and Hematology services. He had not manifested any typical signs or symptoms of secondary hemochromatosis. The Hematology service was consulted, and as per protocol, he was started on twice weekly phlebotomies [7]. Due to pre-syncopal episodes, he was unable to tolerate this, and thus, phlebotomy was performed weekly.

**Table 1:** Iron values

Date	Iron (μmol/L)	Total iron binding capacity (μmol/L)	Saturation index (%)	Ferritin (μg/L)
July 28/2012 (admission)	–	–	–	–
August 2/2012	5	25	20	73
October 30/2012	5	10	50	1883
May 8/2013	4	24	17	>1500
October 19/2013	23	30	77	1425
February 6/2014	11	68	16	
April 30/2014	46	49	94	>1650
May 7/2014	32	47	68	
May 14/2014	38	<41	>93	>1650
May 21/2014	34	<37	>92	>1650
May 23/2014	31	34	91	>1650
May 28/2014	30	34	88	>1650
June 4/2014	35	<38	>92	>1650
June 20/2014 (discharge)	–	–	–	–

Normal values: serum iron (8–25 μmol/L), total iron binding capacity (40–80 μmol/L), saturation index (16–60 %), ferritin (12–300 μg/L)

### Discussion

There is a wide differential of potential causes to consider in patients presenting with iron overload. In this case, the MRI suggested a pattern of iron overload consistent with a secondary (i.e., non-hereditary) process. A review of secondary causes reveals that he did not receive parenteral iron during his stay, did not have a history of chronic anemia or liver disease prior, is not of African descent, and did not have any neurological dysfunction to suggest aceruloplasminemia. Based upon the quantity of RBC transfusions administered during his course, it appears this is the most likely diagnosis.

This is the first reported case of transfusional iron overload resulting in secondary hemochromatosis in a burn patient. Previously, this phenomenon has been shown to occur in patients receiving chronic transfusions over an extended period of time for treatment of blood disorders [8, 9]. This evidence is in keeping with the notion that secondary hemochromatosis due to transfusions is mainly a concern in chronically transfused patients. It has been suggested that signs of iron overload could be present with as few as 10–20 transfusions [10]. This case substantiates that multiple acute transfusions may also lead to secondary hemochromatosis.

Based upon this report, a screening protocol for secondary hemochromatosis may be useful in burn patients receiving acute transfusions, as has been suggested in the population receiving chronic transfusions; however, the details of such a protocol require further study [9]. Screening for secondary hemochromatosis requires straightforward measurement of serum iron indices, and treatment includes iron chelation and/or serial phlebotomy. There is a technique for phlebotomy described in the hereditary hemochromatosis population that could possibly be applied to transfusionally overloaded patients. The suggested regiment includes removing one unit of blood once to twice a week as serum ferritin analysis is performed periodically to ensure its decline [7]. Given the simplicity of screening and availability of treatment options, which reduce long-term morbidity, further research in this area is essential to document the prevalence of secondary hemochromatosis in the burn population and the potential need for such a screening protocol.

## Conclusions

Burn patients treated with RBC transfusions are at risk of iron overload and secondary hemochromatosis. Awareness of this treatable condition can reduce long-term morbidity. Further investigation is required to define screening protocols.

## Consent

Written and informed consent was obtained from the patient for publication of this case report and any accompanying images.

## References

[1] Van Loey NE, Van Son MJ (2003). Psychopathology and psychology problems in patients with burn scars: epidemiology and management. Am J Clin Dermatol.

[2] Al-Mousawi AM, Mecott-Rivera GA, Jeschke MG, Herdon DN (2009). Burn teams and burn centers: the importance of a comprehensive team approach to burn care. Clin Plast Surg.

[3] Palmieri TL, Greenhalgh DG (2004). Blood transfusion in burns: what do we do?. J Burn Care Rehabil.

[4] Fraga GP, Bansal V, Coimbra R (2010). Transfusion of blood products in trauma: an update. J Emerg Med.

[5] Alessandrino EP, Amadori S, Barosi G, Cazzola M, Grossi A, Liberato LN (2002). Evidence- and consensus-based practice guidelines for the therapy of primary myelodysplastic syndromes. A statement from the Italian society of hematology. Haematologica.

[6] Andrews NC (1999). Disorders of iron metabolism. N Engl J Med.

[7] Bacon BR, Adams PC, Kowdley KV, Powell LW, Tavill AS (2011). Diagnosis and management of hemochromatosis: 2011 practice guideline by the American association for the study of liver diseases. Hepatology.

[8] Rotaru I, Gaman A, Gaman G (2014). Secondary haemochromatosis in a patient with thalassemia intermedia. Curr Health Sci J.

[9] Lichtman SM, Attivissimo L, Goldman IS, Schuster MW, Buchbinder A (1999). Secondary hemochromatosis as a long-term complication of the treatment of hematologic malignancies. Am J Hematol.

[10] Porter JB (2001). Practical management of iron overload. Br J Haematol.

